# Potential travel cost saving in urban public-transport networks using smartphone guidance

**DOI:** 10.1371/journal.pone.0197181

**Published:** 2018-05-10

**Authors:** Cuiying Song, Wei Guan, Jihui Ma

**Affiliations:** MOE Key Laboratory of Urban Transportation Complex System Theory and Technology, Beijing Jiaotong University, Beijing, P. R. China; Beihang University, CHINA

## Abstract

Public transport (PT) is a key element in most major cities around the world. With the development of smartphones, available journey planning information is becoming an integral part of the PT system. Each traveler has specific preferences when undertaking a trip, and these preferences can also be reflected on the smartphone. This paper considers transit assignment in urban public-transport networks in which the passengers receive smartphone-based information containing elements that might influence the travel decisions in relation to line loads, as well as passenger benefits, and the paper discusses the transition from the current widespread choosing approach to a personalized decision-making approach based on smartphone information. The approach associated with smartphone guidance that considers passengers’ preference on travel time, waiting time and transfer is proposed in the process of obtaining his/her preferred route from the potential travel routes generated by the Deep First Search (DFS) method. Two other approaches, based on the scenarios reflecting reality, include passengers with access to no real time information, and passengers that only have access to the arrival time at the platform are used as comparisons. For illustration, the same network proposed by Spiess and Florian is utilized on the experiments in an agent-based model. Two experiments are conducted respectively according to whether each passenger’s choosing method is consistent. As expected, the results in the first experiment showed that the travel for consistent passengers with smartphone guidance was clearly shorter and that it can reduce travel time exceeding 15% and weighted cost exceeding 20%, and the average saved time approximated 3.88 minutes per passenger. The second experiment presented that travel cost, as well as cost savings, gradually decreased by employing smartphone guidance, and the maximum cost savings accounted for 14.2% of the total weighted cost.

## Introduction

Public transit systems play an important role in transporting passengers in metropolitan areas [1] and are coping with an increasing demand for the existing transit network, which is currently one of the largest transport challenges. Most transit users generally have origin and destination locations and expected departure or arrival times before starting a transit journey. Based on the accessing information from the provided transit agencies, transit users need to choose travel routes and transfer stations that can appropriately fit their travel needs. However, seeking a suitable travel route manually is complex because it is difficult for a passenger to determine proper transfer points between different routes because of the lack of necessary vehicle real time information, such as the accurate vehicle arrival times because of the fluctuation of traffic speed due to congestion or bad weather. Intelligent Transportation Systems (ITS) and Advanced Public Transport Systems (APTS) are able to provide timely information to transit users on the conditions of the network, such as lines, schedule, arrival time, departure time, occupancy and transfer. This information can be available before trip departure (pre-trip) or during the trip (en-route) and be delivered via a wide variety of media, such as audible or visual messages through at-stop or in-vehicle information devices, the Internet, and through smartphones, which are a widespread communication tool, to individual transit users or users’ groups. Hence, the analysis on the effects of user choices based on different amounts of the real time information available needs to be evaluated to enable passengers to take more adaptive decisions. Several new studies [2–7] discuss the effect of real-time information on passengers’ route choice. In dynamic path choice model, PT users’ travel choices are adapted with the received real time information regarding the next vehicle arrival time [2, 3]. Furthermore, real-time information can also affect the choice of departure time and stop as well as route choice used by PT users [4] who want to save their travel time [5] or maximize their expected utility [6]. More predictive real-time information, especially crowding information on board, can attract more PT users to choose a more comfortable and efficient travel route [7] based on the transit environment [8].

## Literature review

Most conventional transit assignment models (TAM) are static equilibrium assignment models, which are insensitive to service disturbances, the effects of information and incidents. Transit network formulation can be broadly divided into the two classes: frequency-based TAM (FB-TAM) and schedule-based TAM (SB-TAM). This classification is based on the representation of the transit network as it has substantial impacts on the passenger loading procedure. FB-TAM represents the transit network at the line-level with the corresponding frequencies, while SB-TAM includes a more detailed representation of the time-dependent specific vehicle-runs [9].

In the FB-TAMs, a significant improvement in the field of transit path choice is the result of studies by Spiess and Florian [10]. These researchers defined a travel strategy that allows a person to reach his or her destination at minimum expected cost. The travel strategy aims to minimize the total travel time, including waiting, accessing and in-vehicle time. It is still assumed that passengers board on the first arriving bus from the attractive transit line, in which the total actual travel time is no longer than their expected travel time of the remaining lines in the set. De and Enrique [11] defined an alternative method of generating minimum cost routes and dispatching paths to different lines using a common route section by non-linear programming. Kurauchi et al. [12] proposed a new approach to solve the transit network loading problem using an absorbing Markov chain analogy, which incorporates line capacity constraints through failure-to-board probabilities when considering the common lines problem. Furthermore, Schmöcker et al [13] proposed an FB-TAM by introducing a “fail-to-sit” probability in determining passengers’ route choice, concerning the travel cost based on the likelihood of travelling seated or standing. Although FB-TAMs built the foundation for finding solutions for transit assignment in varieties of traffic scenarios, these models can be inappropriate for today’s reality. With the development of information and communication technologies, the highly regular and information-rich properties of advanced transit systems may justify passengers’ clever arrival strategies and it is necessary to model the within-day dynamics in passengers’ route choices. Hence, schedule-based models appear in recent studies on dynamic transit assignment.

SB-TAMs represent both the supply and demand sides of the transit system as time-independent. Transit service is represented in terms of individual vehicle runs following a given timetable. Nuzzolo and Crisalli [14] review the representative SB approaches in dynamic transit modeling. The stochastic assignment procedure shows the sensitivity coefficients attached to the components of generalized cost [15]. Poon et al. [16] provides a dynamic UE transit assignment, also using an SB network with a given time-dependent O-D demand. It is assumed that transit vehicles with capacity constraints operate precisely as scheduled and passengers queue according to FIFO rule. Each vehicle’s available capacity is updated dynamically as demand is loaded onto the network through a time-increment simulation. Passenger arrival-departure profiles at all stations are recorded after each run considering queueing delays. The UE assignment is solved by the method of successive averages (MSA). Moreover, stochastic components to describe the difference in passengers’ preferences construct the passenger utility function to describe the passengers’ route choices [17–18]. Schedule-based models generally use detailed departure or arrival times for each transit vehicle in making assignment decisions. Passengers who are divided into groups distinguished by desired arrival time use travel strategies in an ordered set in time-expanded network [19–20]. However, it may be essential for more precise simulation models that enable the incorporation of multi-user classes and their respective interactions in the transport network along with information provisions and decision processes [21]. Hence, a third assignment model called the “Agent-based” (AB) model is proposed, in which single passengers are simulated and loaded into buses, as in Cats et al [22]. Aggregate results might reflect the schedule-based approach.

The AB model has substantial advantages in the development of dynamic transit assignment models that are practical for realistic networks. The main issues of the AB approach are supply uncertainties and adaptive user decisions; hence, they identified dynamic loading process and multi-agent-based simulations as two potential approaches for modeling complex transit systems. In the AB model, transit users make route choices based on the access of real time information. A multi-agent simulation model of transit passengers was proposed by Meignan et al [23]. Passenger behavior was modeled as a single pre-trip mode choice decision based on the numbers of smart card history data [24]. This decision considered three alternatives: the shortest path by car, walking and transit alternatives. Waiting time was calculated as half the planned headway. This implied that passengers always take the shortest path for a given travel pattern [25], thereby lacking path choice modeling framework. The transit simulation model in some studies [26–27] was designed to support the evaluation of operations planning and control in which a framework for a multi-agent transit operations and assignment model which captured supply uncertainties and adaptive user decisions was illustrated [28–29]. The evolution of transit reliability influences both the performance and real time information schemes and the potential benefits that such information can yield by using BusMezzo, an agent-based simulation model. Real time information can also affect the passengers’ choice of departure time and stops and thus the route choice. Fonzone and Schmocker [5] compared two types of passengers with access to real time information based on the agent-based model. Furthermore, the optimal strategy approach of Spiess and Florian [10] was used as a benchmark. Cats et al [30] designed a dynamic stochastic model in transit systems accounting for the dynamic congestion and crowding effects using an agent-based simulation model identically embedded in a comprehensive framework for project appraisal. Increasingly numerous applications [31] on driverless vehicles modeling on-demand transit transportation system are using AB models, which improves the implements of fast dispatching decisions.

Our agent-based model is used to simulate passengers using three decision-making approaches based on three scenarios. These approaches are, respectively, “First in” without any real-time information, “Only arrival time” which has access to the arrival time at the platform and “Information from smartphone” with access to travel strategies information in relation to each passenger’s preference from a smartphone. The latter two have access to real time information systems, whereas the first one does not. The focus in this paper is to provide a detailed discussion on the impact of the three decision-making approaches on passengers’ decisions, such as departure time, stop choice and route choice built on an illustrative example referring to the same network that Spiess and Florian [10] used to describe the optimal behavior of passengers without access to information. The detailed simulation procedures for the three approaches are illustrated, as well as the two proposed experiments. To the best of our knowledge, few studies on agent-based transit assignment considering passengers’ personalized preferences on smartphone information, as well as capacity constraints, have been reported.

The rest of the paper is organized as follows: Section 3 describes the static transit network and time-expanded network, notation, and the corresponding assumptions. Section 4 presents the details of three decision-making approaches based on the respective scenarios, as well as the simulation procedures. The two corresponding transit assignment results are analyzed in Section 5 followed by a summary of the primary results and a discussion of future research directions.

## Network description and notations

### Transit networks

**Fig 1:**
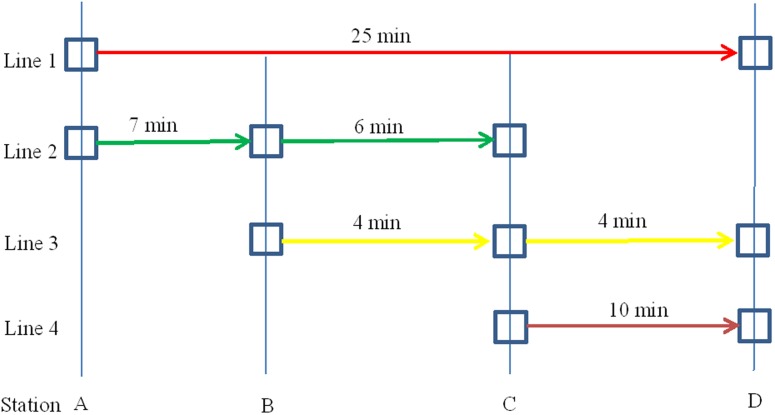
Example of a transit network with four bus lines and four bus stations.

Based on the example network used in the Spiess and Florian [10] paper, a transit network presented in Fig 1 consists of a set of nodes for each run denoting the activity that a passenger may board or disembark at a bus stop served by a line, as well as a set of links connecting associated nodes. The in-vehicle time in a respective line between two nodes for any transit user may not keep constant with the dwell time related to the number of boarding and alighting. The passenger might feel inconveniences, such as not obtaining a seat, crowding on the platform and longer interchanging times at the station. To simplify the calculation complexity, several assumptions in connection with each transit run are given below.

The three situations include (a) no available information accessed by passengers, (b) only the arrival time information displayed on the stop electric information board and (c) complete route information from origin to destination provided by a smartphone. Note that all passengers know approximately the simple transit network information, such as the travel time between two bus stops, the transfer station and the inaccurate frequency through their own commuting experience.

- Transit users in the first two situations are assumed standing at the bus stop; therefore, walking links for the two situations are ignored.
- Traveling fare is not considered as a choosing condition. This assumption seems natural in peak hours for most of the passengers attempting to minimize their total travel time to avoid being late. Furthermore, bus fare is considerably cheaper than other transit modes and has no influence on users’ choice.
- Dwelling times include the time needed for doors to open, boarding and alighting of passengers, closing the doors, and as bus prepares to get off the stop [28]. In the simulation model, the dwell times are determined as a function that relates to the alighting and boarding volumes, as well as the in-vehicle crowding condition. For standard buses, the resulting dwelling time is given by
  $${\text{DT}}_{\text{ijk}}=\text{max}\left(\left(\beta_{1}\text{+}\beta_{3}.\delta_{ijk}^{crowed}\right){.\text{B}}_{ijk}\text{,}\beta_{2}{.\text{A}}_{ijk}\right)+\varepsilon_{ijk}$$ (1)
  where DT_ijk_ = dwell time from line *i* at stop *j* on trip *k*;
  B_*ijk*_ = number of boarding passengers from line *i* at stop *j* on trip *k*;
  A_*ijk*_ = number of alighting passengers from line *i* at stop *j* on trip *k*;
  *β*_1_ = the unit boarding time for per passenger;
  *β*_2_ = the unit alighting time for per passenger;
  *β*_3_ = the unit extra dwelling time for per passenger because of crowding;
  $\delta_{ijk}^{crowded}$ = crowding indicator (= 1 if number of passengers on the bus exceeds the half number of capacity, and 0 otherwise);
  *ε*_*ijk*_ = error term.
- A unitary demand from origin to destination uniformly distributed over 60 minutes has been assigned with a time step of 1 minute. It is common that for each user, he/she clearly knows his/her arrival time at a bus stop, as well as the waiting time.
- Comfort level is determined by boarding on a coming bus or not exceeding the capacity constraints and all passengers satisfying the fail-to-board event.
- The transfer station is on the same position for a passenger’s alighting, that is, there is no need for the passenger to walk to the transfer station after alighting from the previous bus.
- All transit users share the same origin and destination and no passengers board or alight during their travelling.
- Bus capacity is fixed and is related to users’ comfort level and the boarding probability;
- The actual in-vehicle time(or running time) in the simulation process is not fixed, which is given by
  $$IVT_{i,j,j+1,k}^{\text{a}ctual}=IVT_{i,j,j+1,k}^{initial}+\nu_{i,j,j+1,k}$$ (2)
  where $IVT_{i,j,j+1,k}^{\text{a}ctual}$ = the actual in-vehicle time on line *i* from stop *j* to stop *j* + 1 on trip *k*;
  $IVT_{i,j,j+1,k}^{initial}$ = the initial given in-vehicle time on line *i* from stop *j* to stop *j* + 1 on trip *k*;
  *v*_*i*,*j*,*j*+1,*k*_ = the error term.

In Fig 1, four bus lines marked Line 1 to Line 4 are listed with the initial in-vehicle time labelled on the arrow lines. Line 1 connects A and D directly with the longest in-vehicle time, as well as Line 2 to 4, covering transfers stations B and C. Line 3 and Line 4 start from the transfer stations B and C to the destination D with less in-vehicle time.

Associated with each transit line, there is a schedule that lists the departure times at which a transit vehicle must leave its starting station as well as the scheduled arrival time or departure time at stations along its route throughout each day. In our experiments, vehicles passing through stations close to the destination are assumed to depart later in order to ensure the feasibility of passengers’ transfer. All of the departure times on a line depend on the first station’s departure times, the in-vehicle time and the dwelling time. The object for each user is to minimize his/her total traveling time and reduce the associated waiting time. Different from previous research, in which passengers are assigned into routes under the same situation, this assignment method considers a single passenger’s decision under different situations before boarding a vehicle. Each passenger is loaded into buses and the aggregate results might reflect the final results based on the referred approach.

### Time expanded networks

**Fig 2:**
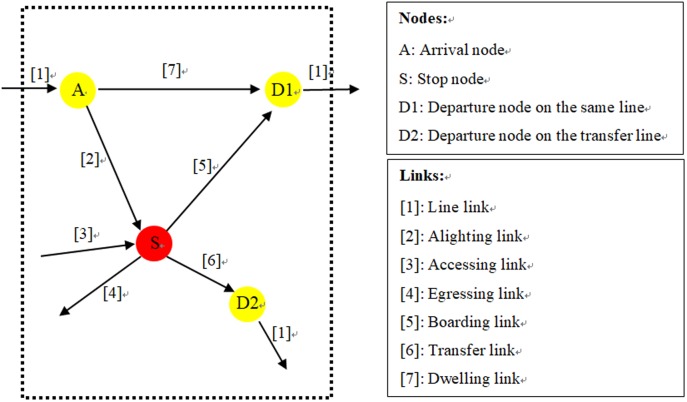
Time expanded network of a single stop for one run.

The model proposed in this study develops a more compact network description (at least for lines with a number of stops) based on line sections, rather than the route section listing all of the time stamps in Hamdouch’s approach. Each node in the time expanded network has two levels: one represents the stop or platform where the event occurs, and the other is a time stamp of the event itself. For each run at a stop, a set of nodes, as well as the connecting links from Fig 2, are generated, representing the arrival and departure events.

The departure times at the first stop for each bus are extracted according to the predefined schedule. The following arrival time at the next stop is determined by the travel time in each segment. However, the departure time, except for the initial stop, depends on the on or off boarding passenger volume. Links connecting nodes represent the passenger flow trends for each run. Beginning with a line link from the previous stop, passengers on board are split into two groups, one for alighting and the other for remaining, corresponding to the alighting link and dwelling link.

For all passengers standing on this stop now just after the running bus, the three flow splits are divided, that is, arriving at the destination, such as at stop D matching the egressing link, transferring to another line, such as at stop B or C, and boarding onto this bus. The departure nodes are also connected by a line link at the beginning of the next stop. Fig 2 shows the typical time expanded network configuration for each run at one stop. Not all nodes and links are needed for each stop; for example, there are no arrival nodes and egressing links on the initial stop. Furthermore, the time expanded network changes depending on the route strategies and on the received real-time information.

## Decision-making approaches

We assume that all the passengers are risk-adverse and that she or he makes a decision in the choice set that potentially minimizes the total travel cost according to the perceived real time information. Three decision-making approaches have been proposed based on the preliminary scenarios. The approach with no real time information in the first scenario is called “First in” (FI), that is, passengers take the first coming vehicle whenever they are standing at the bus stop with no available real time information or only approximate frequency information that is omitted for the risk-adverse passengers. The provided approach called “Only arrival time” (OAT) addresses the second scenario when passengers only know the arriving time at the initial station from the electric board and choose one from several coming vehicles. A third approach considers that the decision maker is supposed to know the schedule of lines in the whole journey by consulting an online smartphone and chooses the preferred one from the obtained choice set, referred to as “Information from smartphone” (IFS). Traditionally, it is assumed that passengers care for real time information only for services with low frequency. However, it seems reasonable to assume that passenger will take advantage of widespread online real time information even if the frequency is generally high when considering transfers, especially through advanced smartphone technology. Furthermore, we assume that the three approaches mentioned before are all Agent-based decision making methods to ensure their comparison under the same line schedule.

### FI method

In the FI method, the passenger does not have access to real time information. Therefore, it is not possible to distinguish between the earliest arrival connections and shortest connections. It is assumed that the decision maker leaves immediately from the origin and adopts the least wait time run and the associated line. However, if the vehicles for both the direct line and transfer line arrive at the same time (at the origin), he/she chooses the direct line first. The potential route strategies about Fig 1 are listed below, from which no passengers from stop A will transfer at stop B to reduce their possibilities to reach the destination. Furthermore, more runs are chosen at stop C with two lines than stop B with only one line if accessing no real time information. However, passengers boarding on the first coming bus in this method to minimize their waiting time may occasionally sacrifice the shortest travel time. Therefore, in this study, this method is set as the comparison with the other two methods.

**Route 1**: Line 1 (A-D);
**Route 2**: Line 2 (A-C) -- Line3 (C-D);
**Route 3**: Line 2 (A-C) -- Line4 (C-D).

### OAT method

The second scenario describes a scenario where one passenger accesses the arrival times of coming buses (generally less than five runs) from an electric information board at the current station. No extra information is provided in advance to determine the arrival time for the next transfer stations if one or more transfers are necessary. After receiving the arrival time information, he/she chooses the preferred route. This finding implies that he/she may choose different routes even when accessing the same information.

**Fig 3:**
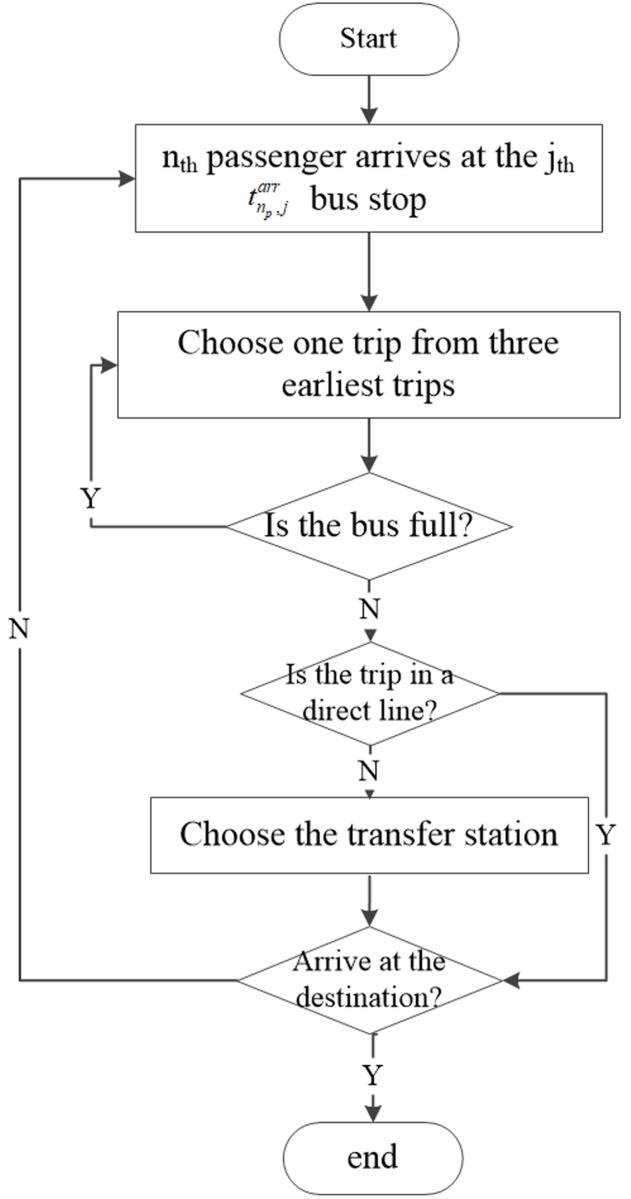
Procedure of OAT method.

An outline of the OAT method for one passenger *n*_*p*_ standing at the *j*_*th*_ bus station is displayed in Fig 3. He/she chooses one trip from the potential bus set *S*_*choose*_*veh*_ constructed by the three earliest boarding trips. If the chosen bus is not a direct line, it is necessary to choose the transfer station before arriving at the destination. The main procedures of determining one’s travel journey are presented below.

#### 1) Roulette method to determine the next coming bus

**Fig 4:**
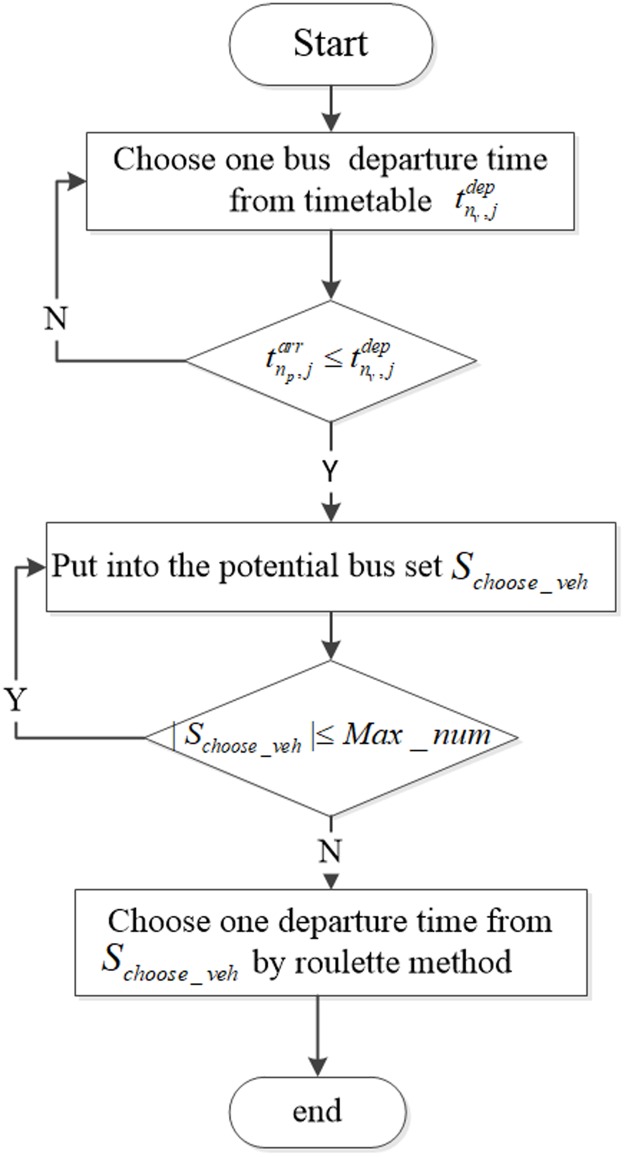
Procedure of choosing departure time using the OAT method.

In this method, we assume that the maximum number of potential trip set is three, and for each vehicle *n*_*v*_, a passenger’s maximum expected waiting time is $T_{w}^{\text{max}}$. We assume that the decision maker possesses the information regarding the next three earliest trips based on his/her expected or actual arrival time $t_{n_{p},j}^{arr}$ at the *j*_*th*_ station. If the departure time $t_{n_{v},j}^{dep}$ for each trip is not more than one’s arrival time, this bus is placed into the potential bus set *S*_*choose*_*veh*_, seen in Fig 4, and the passenger attempts to board the bus. Thus, the choosing probability $P_{n_{v},j}^{dep}$ for each vehicle *n*_*v*_ is formulated as

$$P_{n_{v},j}^{dep}=\{\begin{array}{cc} \frac{T_{w}^{\text{max}}-(t_{n_{v},j}^{dep}-t_{n_{p},j}^{arr})}{\sum_{n_{v}\in S_{choose\_veh}}T_{w}^{\text{max}}-(t_{n_{v},j}^{dep}-t_{n_{p},j}^{arr})} & T_{w}^{\text{max}}\geq (t_{n_{v},j}^{dep}-t_{n_{p},j}^{arr})\\ \frac{0.1}{\sum_{n_{v}\in S_{choose\_veh}}T_{w}^{\text{max}}-(t_{n_{v},j}^{dep}-t_{n_{p},j}^{arr})} & T_{w}^{\text{max}}<(t_{n_{v},j}^{dep}-t_{n_{p},j}^{arr}) \end{array}$$ (3)

The choosing probability $P_{n_{v},j}^{dep}$ closely associates with $T_{w}^{\text{max}}$. If one passenger’s waiting time is lower than $T_{w}^{\text{max}}$, $P_{n_{v},j}^{dep}$ entails a higher value to enlarge the selected probability. In contrast, the preliminary numerator formula is replaced by a relatively small value (0.1 in this instance). Next, in the roulette method, one trip is randomly chosen based on the respective proportion probability $P_{n_{v},j}^{rou}$.

$$P_{n_{v},j}^{rou}=\frac{P_{n_{v},j}^{dep}}{\sum_{n_{v}\in S_{choose\_veh}}P_{n_{v},j}^{dep}}$$ (4)

$P_{n_{v},j}^{rou}$: Proportion probability for vehicle *n*_*v*_ at the *j*_*th*_ station.

#### 2) Determine the transfer station

Once a passenger determines the boarding vehicle, then it is essential for him/her to choose the transfer station or next alighting station if the boarding trip is not on a direct line. In this case, the dominant rule is to reduce the transfer times. If the minimum transfer times are the same for some transit routes, we randomly choose one station from the potential transfer station set. For example, in Fig 1 for the departure trips from line 2, three transit routes presented below transfer for once. One transfer station is stochastically retrieved from the potential transfer station set consisting of station B and station C.

**Route 1**: Line 2 (A-B) -- Line3 (B-D);
**Route 2**: Line 2 (A-C) -- Line3 (C-D);
**Route 3**: Line 2 (A-C) -- Line4 (C-D).

### IFS method

**Fig 5:**
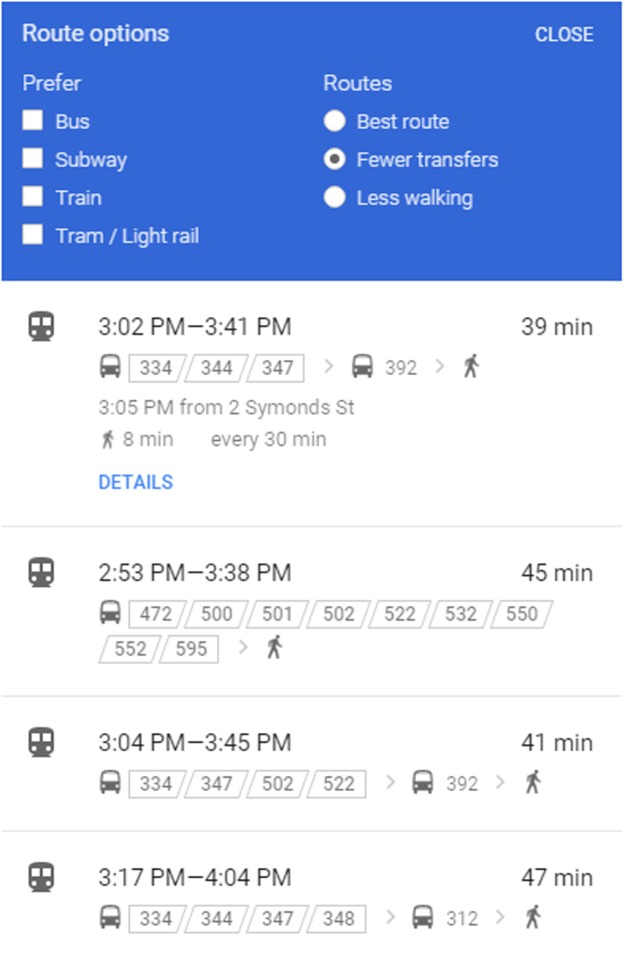
Real time information on fewer transfers option from Google Maps.

**Fig 6:**
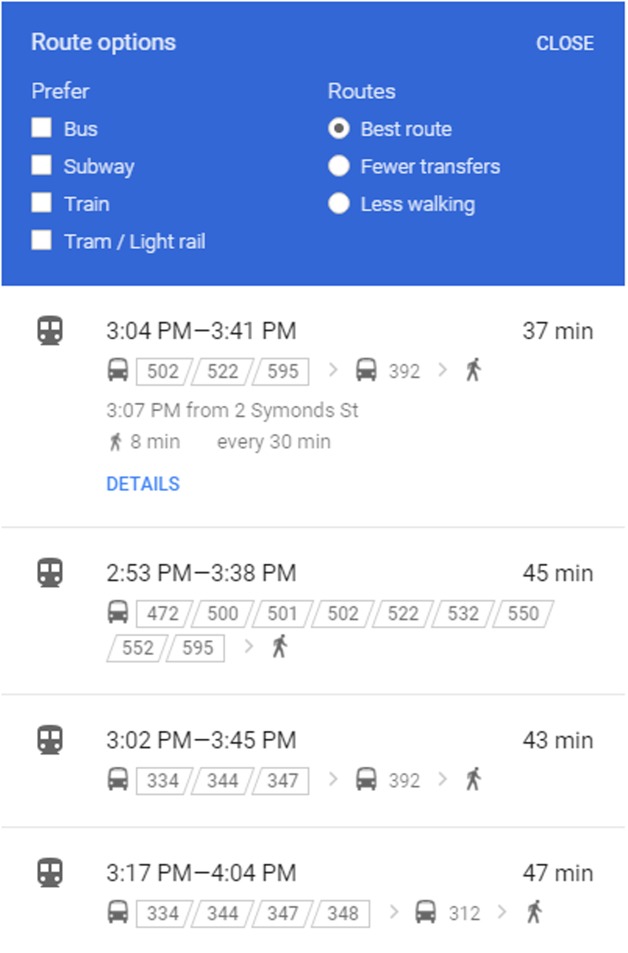
Real time information on best route option from Google Maps.

The decision-maker has the information concerning a list of potential travel routes, illustrated in next section, from all possible lines (in our network, all buses directly or indirectly connected to the destination) by searching from their mobile device, allowing them to know the different route options and benefits, such as less transfers from Fig 5, shorter travel distance, less waiting time, less walking and best route from Fig 6. The records of routes consist of lines and the related boarding or alighting stations from origin to destination represented on the mobile device. The decision-maker chooses one of them as his/her best route. The whole chosen routes generated by the Deep First Search (DFS) method based on Fig 1 are described below:

**Route 1**: Line 1 (A-D);
**Route 2**: Line 2 (A-B) -- Line3 (B-D);
**Route 3**: Line 2 (A-C) -- Line3 (C-D);
**Route 4**: Line 2 (A-B) -- Line3 (B-C) -- Line4 (C-D);
**Route 5**: Line 2 (A-C) -- Line4 (C-D).

**Fig 7:**
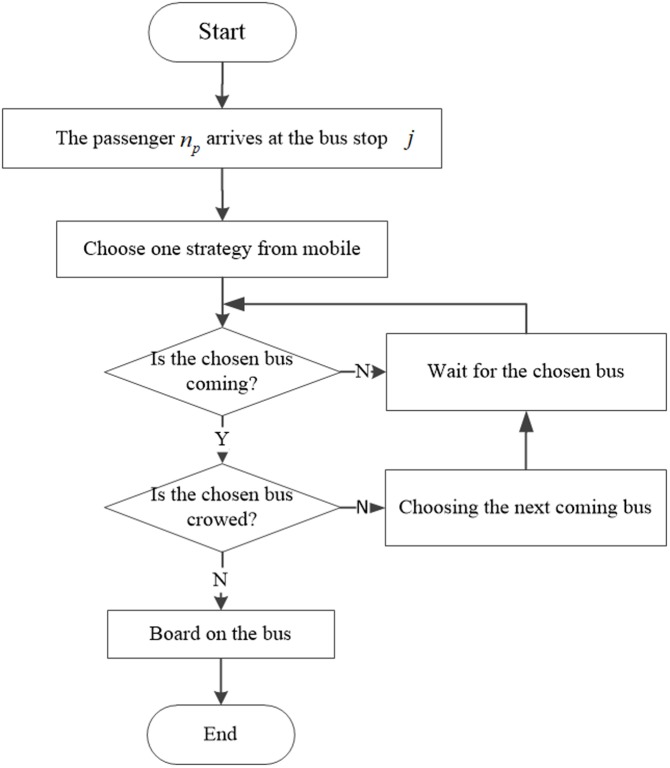
Passengers’ travel procedures using real time information on a smartphone.

**Fig 8:**
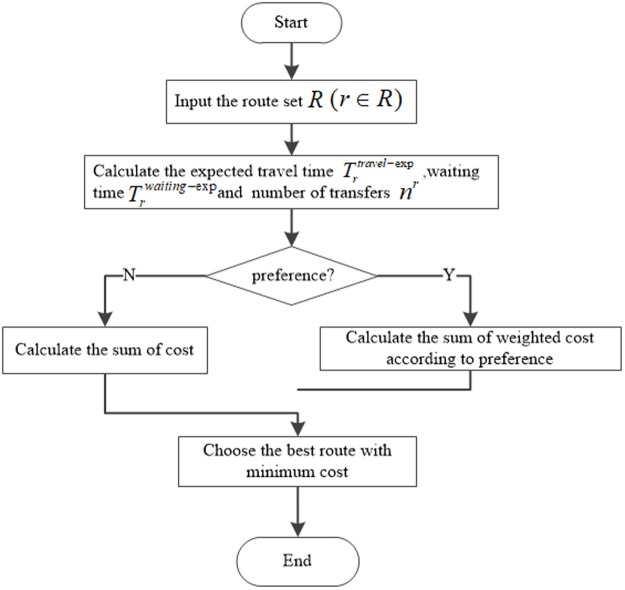
Procedure of generating optimal routes displayed on smartphone.

The generating procedures of the detailed travel strategy are shown on Fig 7. After inputting the current location (based on the Global Positon System (GPS)) and a passenger’s expected arrival time ${\text{t}}_{n_{p},j}^{arr}$ at the origin station, he/she acquires a list of routes from the mobile device, chooses the best one according to his/her travel preference as the objective strategy, and waits for the first bus displayed in the strategy. Because of capacity or congestion constraints, he or she has to decline the currently incoming bus if the objective bus is quite crowed. Two main procedures are divided based on the two key factors seen in Fig 7. For one passenger preparing to travel or just arriving at the bus stop, we assume that he/she has no travel strategy before searching on the mobile device widely used by transit users and that the search is the only way to receive travel information in this method. When a passenger arrives at the bus stop at the arrival time ${\text{t}}_{n_{p},j}^{arr}$, he/she chooses the optimal route as the current strategy by searching from a smartphone. Next, he/she waits for the arrival of the first bus on the chosen route. If the chosen bus arrives later than the passenger at the current stop *j*, he/she has to wait. If the chosen bus is crowded such that it has no seats or insufficient capacity to board, he/she has to wait for the next coming bus. The procedure for generating the optimal strategy associated with one’s preference is presented on Fig 8. The calculation process of each respective cost attribute, including expected travel time, expected waiting time and number of transfers, begins after the input of the set route. Then, the weighted cost based on the related passenger’s preferences is computed before choosing the best route with a minimal cost value. In this instance, the expected waiting time is calculated with the consideration of the maximum dwelling time.

The weighted route travel cost associated with each parameter, is defined by

$$d_{\text{r}}=\sum_{m}\alpha^{m}c_{r}^{m}\;\;\;\forall \text{r}\in R$$ (5)

where $c_{\text{r}}^{m}$ is the cost attribute *m* associated with route *r* and *α*^*m*^ is the weighting parameter applied to attribute *m* based on users’ preferences.

The cost of transfer, per passenger, is assumed to be 1.0 ¥ for one transfer and the values of waiting time and travel time are set 0.35 ¥/min and 0.24 ¥/min, respectively. The base values of different attributes associated with the three path options, along with unweighted path costs, were computed in Table 1.

**Table 1:** Numerical example base values.

	Travel		Waiting		Transfer		Route cost
	Value (mins)	Value (¥)	Value (mins)	Value (¥)	Value (¥)	Value (¥)	Value (¥)
**Route 1**	25	6	10	3.5	0	0	**9.5**
**Route 2**	15	3.6	20	7	1	1	11.6
**Route 3**	17	4.08	17	5.95	1	1	11.03
**Route 4**	21	5.04	17	5.95	2	2	12.99
**Route 5**	23	5.52	15	5.25	1	1	11.77

Table 2 lists the path recommendations for three types of travelers with different preferences. Traveler 1 weights all the preferences equally, while passengers 2 and 3 dislike waiting and traveling for long periods of time, respectively. For passengers 1 and 2, the best route is Route 1, while for passenger 2 it is Route 2. This result demonstrates that different passenger’s preferences lead to a different order of routes. Clearly, more complex travel strategy methods are conceivable and could be generated by various cost functions. For example, it is likely that passengers will consider any or all of the following: fares, seat availability, and different decision processes such as the consideration of real time information in connection with previous experience. Furthermore, decisions may be subject to particular constraints, such as that some passengers might be captive to a subset of lines and specific departure times. Such constraints may reduce the impact of information provided by a smartphone. For example, passengers aim to minimize their travel times, but if they can use only certain lines, they will have fewer chances to select a trip that is different from what they could choose with real time information from a smartphone.

**Table 2:** Route recommendations for travelers with different preferences.

	Value of Weighting Parameters				Weighted Path Cost (¥)		
	Traveler 1	Traveler 2	Traveler 3	Route No.	Traveler 1	Traveler 2	Traveler 3
*α*^*wait*^	1/3	**0.6**	0.2	**Route 1**	**3.17**	**3.3**	4.3
*α*^*travel*^	1/3	0.2	**0.6**	**Route 2**	3.87	5.12	**3.76**
*α*^*transfer*^	1/3	0.2	0.2	**Route 3**	3.68	6.586	3.838
				**Route 4**	4.33	4.978	4.614
				**Route 5**	3.92	4.454	4.562
**Route recommendation**					1,3,2,5,4	1,5,4,2,3	2,3,1,5,4

## Experiments and results

Six thousand simulation runs were performed for the three mentioned methods. In every run, the distribution of the demand among different lines is calculated under each decision-making approach. A fixed demand *N* (200 in this experiment) of passengers from Stop A to Stop D in Fig 1, uniformly distributed over 3600 seconds (60 minutes), has been assigned with a time step of 1 second. Two experiments associated with the same condition have been performed: 1) the experiment associated with the three decision-making methods is conducted respectively based on consistent passengers; 2) an experiment related to passengers inconsistent with their decision-making methods is implemented. The simulations were run with MATLAB 2015b on an Intel Core(TM) i7-6500 CPU at 2.50 GHz with 8.00GB of RAM. The timetable was given in Table 3 according to the initial departure stop of the given lines in Fig 1. Departure times of the passing stops were calculated depending on the in-vehicle time depicted between two stops of the respective line in Fig 1, and the dwell time was calculated by Formula (1). In addition, the limited capacity C for each vehicle is set as 20 and the weighting parameter is set as 0.7 for the cost preference attribute. *β*_1_, *β*_2_ and *β*_3_ are set as 4, 2 and 4 seconds, respectively. The maximum transfer number is set as 3, the dwell time error term *ε*_*ijk*_ is randomly obtained, ranging from 0 to 20 seconds, and the error term of in-vehicle time *v*_*i*,*j*,*j*+1,*k*_ ranges from 0 to 120 seconds (2 minutes). The preferences of each passenger are randomly generated, and the number of simulation runs is set as 6000.

**Table 3:** Timetable based on the initial departure stop of the respective line illustrated in Fig 1.

Vehicle	1	2	3	4	5	6	7	8	9	10	11	12	13	14	15	16	17
**Line1**	0	7	14	21	28	33	38	43	50	58	66	74	82	90	98	106	114
**Line2**	1	6	11	16	21	25	29	33	37	43	50	57	64	71	78	85	92
**Line3**	8	13	18	23	28	34	40	46	52	60	68	76	84	92	100	108	116
**Line4**	12	15	18	22	26	30	34	38	42	47	52	57	63	69	75	81	87

### Experiments for passengers with consistent choosing method

Table 4 illustrates the results incorporating total travel time, waiting time of the whole journey, saved time and waiting time at the origin stop of the experiments as well as the weighted cost based on the three mentioned methods. One passenger’s travel time is calculated from the time elapsed between his/her arrival at the origin to his/her arrival at the destination, incorporating waiting time, in-vehicle time and dwelling time. Compared with the aforementioned FI and OAT methods, the IFS methods can reduce travel time by approximately 18.4% and 15.3%, respectively. The total waiting time of the whole journey for the IFS method is relatively lower than the other two methods due to a decreased waiting time at the origin stop. The difference between the time at which the passenger consults the information and that at which he/she leaves the origin is called saved time. For the passenger accessing information by smartphone, the waiting time at the origin is regarded as zero if the first vehicle in the optimal route is available. Therefore, the saved time can also be described as the unnecessary waiting time. The average saved time in this case approximates to 3.88 minutes per passenger. The total waiting time at the origin stop for IFS methods is evidently lower than for other methods due to the utilization of real time information from the smartphone. The OAT method has the least total number of transfers. The weighted cost for IFS is decreased by 23% and 26.5% compared with FI and OAT methods, respectively.

**Table 4:** Results of experiments for passengers with the same preference.

Method	Total travel time (minutes)	Total waiting time of the whole journey(minutes)	Total saved time (minutes)	Total waiting time at origin stops (minutes)	Total number of transfer	Weighted cost (¥)
**FI**	5649.48	1479.48	0	777.48	160	571.2161
**OAT**	5441.9	1138.85	0	730.05	104	597.8661
**IFS**	4609.57	1359.38	776.85	19.47	119	439.3741

**Fig 9:**
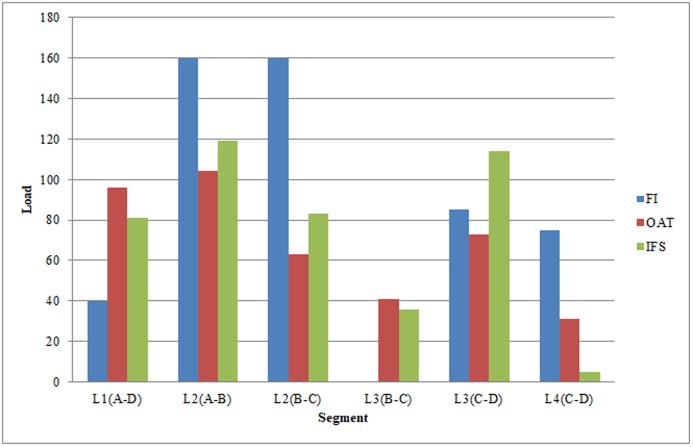
Assignment results of load per segment.

**Fig 10:**
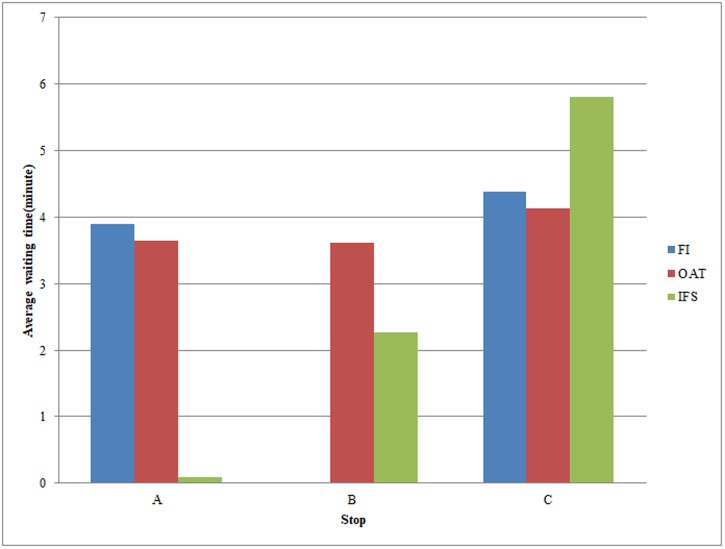
Assignment results of average waiting time per stop.

Figs 9 and 10 illustrate the specific assignment results on line usage. In the upper part of the figure the bars represent the passenger load for each segment derived from the divided bus lines by the middle stops. The bottom part of figure shows the average waiting time for passengers at the stops. This stop-related information can be useful for public transportation operators, who may use it to decide where to locate their services. In Fig 9, the IFS method aims to save passengers’ waiting time, as well as minimizing the travel time without delaying arrival time excessively. In comparison with the network usage in FI method, passengers under IFS migrate from L4 to L3. In particular, the IFS method induces a reduction of 93% of L3 compared to the FI method. The usage of L3 is much less under the FI method, but it becomes the second-most-used line under the IFS method. In addition, in the FI method, the segment L3 at stop B is not used at all, whereas under IFS, nearly one fifth of passengers travelling from L2 at stop B transfer to L3, which is faster than the segment on line L2. At stop C, the ratio of the shares on the two segments is balanced under the FI method, whereas there are a large proportion of passengers changing from L4 to L3 because of the tendency to prefer fast lines induced by the availability of information. In general, under the OAT method, the use of lines is in between the usage observed with the FI and IFS. Similar to IFS, OAT passengers tend to shift to faster lines as a consequence of the availability of information, but their tendency to reduce the travel time is limited by the preference of specific lines, such as the direct line.

In Fig 10, clearly stop A is used by all passengers in all of the decision-making methods. The maximum average waiting time at stop A for the FI method is the highest among the above three methods, but there is less waiting time for the IFS methods because in those strategies, passengers are assumed to arrive at the departure stop at the very last moment. The difference of utilizing lines is mirrored in a different use of Stop B. The average waiting time for passengers in the OAT method is the highest. In addition, the transfer frequency is relatively high at stop C involving all passengers using indirect lines, and the average waiting time at stop C is the highest under IFS. This finding implies that more passengers attempt to wait for the shorter route, even though they may spend considerably more time waiting.

### Experiments for passengers with inconsistent choosing methods

This experiment is proposed in order to compare changes from the FI method, which is widely used in real life, to the IFS method, which may become more popular in the future. In Table 5, passengers are divided into five groups based on ratio *γ*^*FI*^ from high to low. The ratio *γ*^*OAT*^ of passengers using the OAT method is set as 0.1, a small proportion compared with other two methods. The ratio of passengers using three choosing approaches is defined as

$$\gamma^{s}=\frac{N^{s}}{N}(s\in S=\{FI,OAT,IFS\})$$ (6)

**Table 5:** Ratio and the related volume of passengers using three choosing methods (*N* = 200).

FI		OAT		IFS	
*γ*^*FI*^	*N*^*FI*^	*γ*^*OAT*^	*N*^*OAT*^	*γ*^*IFS*^	*N*^*IFS*^
0.8	160	0.1	20	0.1	20
0.6	120	0.1	20	0.3	60
0.4	80	0.1	20	0.5	100
0.2	40	0.1	20	0.7	140
0.1	20	0.1	20	0.8	160

**Fig 11:**
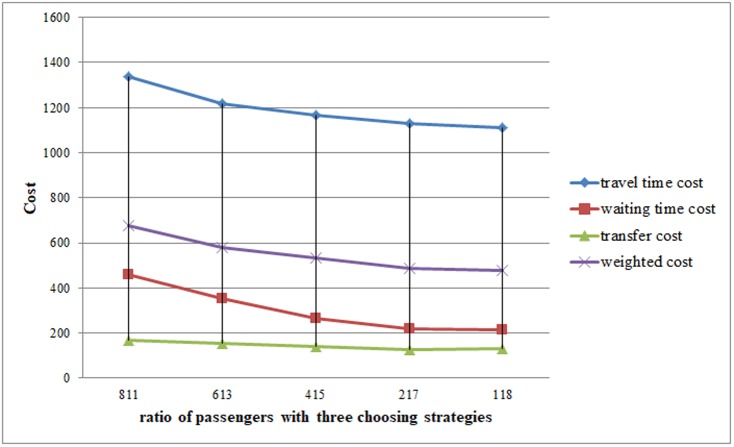
Results of experiments with different ratio of passenger under three choosing methods (811 in horizontal axis is defined as *γ*^*FI*^ = 0.8, *γ*^*OAT*^ = 0.1, *γ*^*IFS*^ = 0.1).

In Fig 11, four types of cost are displayed, including travel time cost, waiting time cost, transfer cost and weighted cost. Clearly, when most passengers choose their travel routes using the FI method, their travel costs are considerably higher. In contrast, the entire costs reduce under the IFS method, which involves the dissemination of real-time information presented by a smartphone. In addition, weighted cost savings gradually decline, and the largest one, 96.23048, takes up 14.2% when the proportion of passengers using the FI method changes from 80% to 60%. This finding implies that passengers’ trips can be improved efficiently in real life using the smartphone-provided travel strategies when the FI method is used by most passengers. However, when the ratio of passengers utilizing smartphone reaches a certain point, cost savings are fewer and even negligible. Furthermore, the travel time weighted cost is the highest and takes up approximately half of total weighted cost. It is reasonable that most passengers pay more attention to travel time, particularly during peak hours.

## Conclusions

This study described the effect of real time information from a smartphone on the passenger’s saved costs. Passengers were able to access information through three methods, which were having access to no travel information, the vehicle arrival information at the platform, and travel route strategies provided by a smartphone to minimize the travel time from origin to destination. Passengers accessing no travel information normally take the first arriving vehicle in our decision making approaches. In the second scenario, where only the arrival information of the vehicles is available, passengers choose their travel methods depending on their preference, simulated by a Roulette and Stochastic method to decide which bus to take and which stop to disembark at, respectively Instead of choosing routes only based on travel time, a more comprehensive method that considers passengers’ preference on travel time, waiting time and number of transfer is designed in the process of generating his/her preferred strategy from the potential travel routes generated by the DFS method in the third scenario. In the experiments, four bus lines with four bus stops are represented in a static transit network and share the same origin and destination, as well as time expanded networks that are designed to describe the process of a passenger’s ride. Two experiments are provided based on whether each passenger’s method of choice is consistent. The results in the first experiment show that even in relatively simple networks, different combinations of providing travel information and approaches lead to significantly different solutions. It is observed that real time information accessed from a smartphone can reduce travel times by more than 15% and save approximating 3.88 minutes per passenger. Furthermore, the total waiting time decreases to a relatively low value leading to an improved reliability of the transport system services and the satisfaction of passengers. From a network management perspective, it is concluded that loads can differ significantly depending on the available information and passenger strategy. Evidently, accessing real time information from a smartphone makes the network more reliable and efficient when it is broadly used. The second experiment illustrates the cost changes or savings when passengers decide their travel using the FI method widely used in real life, compared to the IFS method provided using a smartphone. The result shows that the IFS method can notably reduce travel cost and that cost savings gradually decline when passengers widely choose the FI method over the IFS method. The maximum cost savings reach 96.23048, accounting for 14.2% of the weighted cost, when 80% of passengers choose the FI method.

The present study can be extended in a number of ways. Limitations, such as the missing consideration of ignoring the in-vehicle state information of the coming buses, have already been mentioned. Furthermore, comfort level is only determined by whether the incoming bus has reached capacity. The current study describes the saved time and the related costs when only accessing route information from a smartphone. Further research is also warranted to investigate the effect of newly added information accessed in-vehicle, such as changed arrival times and dynamic in-transit times. In particular, larger networks related to the real-world cases should be analyzed. Additionally, different user cost functions referring to more preferences, such as walking time and travel fare, should be explored in connection with the journey planning strategies. By obtaining actual data when passengers make choices on generating travel strategies, one might obtain more information regarding the actual strategies that passengers use.

## Supporting information

S1 Table Passengers’ arrival origin time. (XLSX)

S2 Table Passengers’ travel preferences. (XLSX)
